# Structure and ligand binding of As-p18, an extracellular fatty acid binding protein from the eggs of a parasitic nematode

**DOI:** 10.1042/BSR20191292

**Published:** 2019-07-23

**Authors:** Marina Ibáñez-Shimabukuro, M. Florencia Rey-Burusco, Mads Gabrielsen, Gisela R. Franchini, Alan Riboldi-Tunnicliffe, Andrew J. Roe, Kate Griffiths, Alan Cooper, Betina Córsico, Malcolm W. Kennedy, Brian O. Smith

**Affiliations:** 1 Instituto de Investigaciones Bioquímicas de La Plata, CONICET-UNLP, Facultad de Ciencias Médicas, calles 60 y 120, 1900-La Plata, Argentina; 2 Institute of Infection, Immunity and Inflammation, University of Glasgow, Glasgow G12 8QQ, UK; 3 Australian Synchrotron, Clayton, Victoria, Australia; 4 Institute of Biodiversity, Animal Health and Comparative Medicine, University of Glasgow, G12 8QQ, UK; 5 School of Chemistry, University of Glasgow, Glasgow G12 8QQ, UK; 6 Institute of Molecular, Cell and Systems Biology, University of Glasgow, Glasgow G12 8QQ, UK

**Keywords:** Ascaris suum, As-p18, fatty acid binding protein, nematode, nemFABP, parasite

## Abstract

Intracellular lipid-binding proteins (iLBPs) of the fatty acid-binding protein (FABP) family of animals transport, mainly fatty acids or retinoids, are confined to the cytosol and have highly similar 3D structures. In contrast, nematodes possess fatty acid-binding proteins (nemFABPs) that are secreted into the perivitelline fluid surrounding their developing embryos. We report structures of As-p18, a nemFABP of the large intestinal roundworm *Ascaris suum*, with ligand bound, determined using X-ray crystallography and nuclear magnetic resonance spectroscopy. In common with other FABPs, As-p18 comprises a ten β-strand barrel capped by two short α-helices, with the carboxylate head group of oleate tethered in the interior of the protein. However, As-p18 exhibits two distinctive longer loops amongst β-strands not previously seen in a FABP. One of these is adjacent to the presumed ligand entry portal, so it may help to target the protein for efficient loading or unloading of ligand. The second, larger loop is at the opposite end of the molecule and has no equivalent in any iLBP structure yet determined. As-p18 preferentially binds a single 18-carbon fatty acid ligand in its central cavity but in an orientation that differs from iLBPs. The unusual structural features of nemFABPs may relate to resourcing of developing embryos of nematodes.

## Introduction

Intracellular lipid binding proteins (iLBPs) are a large family of small, ∼15 kDa, cytosolic proteins that include the fatty acid-binding proteins (FABPs) and the retinol- and retinoic acid-binding proteins (CRBPs, CRABPs) [1]. The nine human FABP isoforms are thought to expedite the transport of sparingly soluble, membrane-damaging, and chemically sensitive fatty acids and their metabolites within the cytosol [2]. Several iLBPs have been shown to be involved in cellular regulation, such as in the mobilization of lipid stores in mammalian adipocytes [3] and fat metabolism in nematode worms [4]. Some FABPs are involved in the activation of nuclear receptors that induce transcription of multiple genes encoding proteins involved in fatty acid and glucose metabolism, as well as cell differentiation [5–7], and this appears to be an important function of retinoid-transporting CRBPs and CRABPs [8]. iLBPs have diverse amino acid sequences, but their 3D structures are highly similar, all comprising a 10-stranded β-barrel with two small α-helices forming a cap over the presumed portal for entry and exit of ligands [1].

Intracellular proteins with almost identical folds to the mammalian iLBPs have been described widely amongst vertebrates and across the metazoan phyla, including insects, crustaceans and arachnids [9,10]. This family of proteins appears to be confined to metazoans, and has not yet been reported from plants, protists, bacteria or archaea. iLBPs are translated from mRNAs that do not encode identifiable secretory signal peptides at their N- termini, so are not expected to be released from the synthesizing cell, although there is recent evidence that one isoform at least in mammals is released by a non-conventional secretory pathway and can then act as an adipokine [11]. It is also reported for trematode and cestode parasites that FABPs are released as excretory/secretory products in extracellular vesicles and possess immunomodulatory properties [12–14]. The only exceptions to these rules are proteins of a subfamily of LBPs found in nematodes, the nemFABPs [15] and secretory abundant heat-soluble (SAHS) proteins of tardigrades [16], which possess cleavable secretion signal leader peptides and are secreted from the cell. The first of these to be described from nematodes were As-p18 from the large intestinal roundworm, *Ascaris suum* and Bm-FAB-1 from the agent of lymphatic filariasis, *Brugia malayi* [17]. As well as being produced by these pathogenic nematode parasites of humans and animals, similar, presumed extracellular FABPs have been found encoded within the genomes of other parasites of humans (see below), and in several plant-parasitic nematodes [18,19]. These proteins’ expression patterns are gender-specific and developmentally regulated and some are found in the perivitelline fluid surrounding the developing embryos of the nematodes *Ascaris suum* and *Brugia malayi* [15,17,20–22]. The genome of the free-living nematode *Caenorhabditis elegans* also encodes several FABPs, three of which are developmentally regulated, possess secretory signal peptides and are secreted from cells. One of these, Ce-LBP-1, appears only to be synthesized and secreted by developing embryos within the egg [22], as are nemFABPs of parasitic nematodes [15,17,22]. A further distinguishing feature of nemFABPs is that the primary sequences of the mature proteins are typically 10–19 residues longer than other iLBPs, and in a previous modeling attempt it was proposed that some of these additional residues are accommodated in extended loops [15].

The nemFABPs as a subfamily of iLBPs therefore appear to be confined to nematodes, and are distinguished by being structurally distinct, secreted from cells by a conventional (secretory leader peptide-mediated) pathway, and may be associated with particular functions in nematode reproduction and development. Here we present the structure of a nemFABP, As-p18, from *A. suum*, a close relative of the large intestinal roundworm of humans, *Ascaris lumbricoides*. The structures were determined by both X-ray crystallography and nuclear magnetic resonance spectroscopy (NMR) revealing the nemFABPs’ unique structural features and mode of ligand binding. We find that this protein, and probably all nemFABPs, has a fold similar to that of other iLBPs, but that it possesses two extended loops so far found definitively only in nemFABPs, with the largest of these loops being remote from the presumed portal of entry for ligands and the binding site itself.

## Materials and methods

### Expression and purification of As-p18

The protein was expressed in *Escherichia coli* BL21 (DE3), harbouring plasmids pREP4 and pQE-30/As-p18 [15], which encode the protein with an N-terminal His-tag. For crystallization, cells were grown in LB media and As-p18 was purified to homogeneity by nickel-affinity and size-exclusion chromatographies in a Tris buffer as previously described [23,24].

For NMR studies, unlabelled, ^15^N-labelled and ^13^C^15^N-labelled proteins were expressed in cells grown in M9 minimal medium supplemented with ^(15)^NH_4_Cl and ([^13^C_6_]-)glucose as the sole nitrogen and carbon sources. Recombinant As-p18 was purified as described in [25]. After nickel-affinity and size-exclusion chromatographies, the removal of co-purifying ligands derived from the bacterial expression system was achieved by reverse-phase high-pressure liquid chromatography (RP-HPLC) on a C_8_-silica column equilibrated with 10% (v/v) acetonitrile in water with TFA 0.1% (v/v). The protein was eluted using a segmented linear gradient: 10–30%, 30–70% and 70–100% acetonitrile in the ratio 5:13:2. Delipidation was confirmed by thin layer chromatography (Figure 2A), GC-MS of extracted lipids and by NMR spectroscopy.

For the analysis of bound ligands, cells were grown to an OD_600_ of 0.8–1.0 100 ml LB media per 1 L conical flask at 37°C and 250 revolutions per minute (rpm) before protein expression was induced and the cultures were harvested 3 h later. In these better oxygenated cultures, a higher yield of protein (∼40 mg l^−1^ culture) was achieved.

### Sequence analysis and comparisons

Putative homologues of As-p18 (AAA98565.1) were retrieved by a BLAST [26] search of the protein NCBI non-redundant protein sequence database and supplemented with the putative Ov-FAB1 from NEMBASE (www.nematodes.org accession BF727540). The FABPs included in the alignment were human (Hs-LFABP and Hs-IFABP), rat (Rn-IBABP, Rn-CRBPI and Rn-IFABP), mouse (Mm-CRABPI and Mm-AFABP) as well as other mammalian, bird, amphibian and insect proteins (Bt-HFABP, Ss-ILBP, Gg-LbFABP, Xl- CRABPII, Lm-MFABP and MFB2). Nematode sequences included those from *Caenorhabditis elegans* (Ce-LBP1-3), *Ascaris suum* (As-p18), *Brugia malayi* (Bm-FAB1) and *Onchocerca volvulus* (Ov-FAB1) while sequences from non-nematode parasites included those from the cestodes (tapeworms), *Taenia solium* (Ts-MFABP1-2) and *Echinococcus granulosus* (Eg-FABP1), the trematodes (blood and liver flukes) *Schistosoma mansoni* (Sm14) and *Fasciola hepatica* (Fh15). Previously unidentified secretion leader peptides for the nematode proteins were predicted using Signal-3L (http://www.csbio.sjtu.edu.cn/bioinf/Signal-3L/) and excluded from the sequence alignment. The amino acid sequence alignment and average distance dendrogram were generated using MUSCLE [27] with default parameter values and Jalview [28], respectively.

### Analysis of As-p18-bound fatty acids

Total lipids were extracted using CHCl_3_:CH_3_OH mixture (2:1) from holo-As-p18 (approximately 20 mg of non-RP-HPLC purified protein in approximately 2 ml of phosphate-buffered saline solution (PBS)), apo-As-p18 (approximately 7 mg of RP-HPLC purified protein in 5 ml of PBS) or *E. coli* cells lysed by sonication (approximately 2 ml). As a control for lipid contaminants, an equal volume of PBS was used. To assess the lipid types that co-purify with As-p18, the lipid extracts were analysed by thin layer chromatography (TLC) on Si250 plates (J.T. Baker) and resolved with hexane/diethyl ether/acetic acid (80:20:1, by volume) as previously described [29].

The composition of the As-p18-bound fatty acids was analysed by gas chromatography (GC) of their methyl ester derivatives methylated with BF_3_-methanol as described previously [30]. The individual FA methyl esters peaks were identified by comparing their retention times with those of standards and of relative position to established compounds in literature when a standard was not available [31]. The relative peak area was calculated for each fatty acid methyl ester (FAME) in each chromatogram. The identity of the 18:1 fatty acid co-purifying with As-p18 from *E. coli* was confirmed by GC-MS of its FAME on a DB-35ms GC column (Agilent J&W) with helium as the mobile phase, in comparison with FAMEs produced from oleic acid and vaccenic acid standards. Samples were injected at 250°C, maintained at 50°C for 1 min and then eluted by a temperature gradient to 300°C at a rate of 10°C min^−1^.

### Fluorescence spectroscopy

Fluorescence data were recorded at 25°C and corrected for Raman and background scattering. The fluorescent fatty acid analogue, 11-(dansylamino)undecanoic acid (DAUDA) and the unmodified fatty acids were obtained from Molecular Probes and Sigma, respectively. As-p18 stock solution concentrations were estimated from measurements of absorbance at 280 nm and the protein’s theoretical extinction coefficient. Stock solutions were diluted to give 1.25 μM As-p18 in the cuvette. DAUDA ethanol solution was diluted 1:10,000 in PBS for use in the assays at 1 μM. Stock solutions of all the non-fluorescent competitors were made to approximately 10 mM in ethanol, then diluted 1:100 in PBS for use in the assays. The fatty acid preference of As-p18 was tested by displacement of DAUDA with 0.49 μM competitor. After incubation for 3 min, samples were excited at 345 nm and the DAUDA fluorescence was recorded at the peak fluorescence emission wavelength of DAUDA in the protein (500 nm) [18]. For each condition, at least two replicates were done.

### Isothermal titration calorimetry

Thermodynamic parameters for binding of oleic acid to As-p18 were measured using a MicroCal VP-ITC (1.4 ml working volume; 320 rpm stirring; 25°C), and all solutions were degassed briefly before use [32]. Each experiment involved 28 sequential 10 μl injections of 0.325 mM oleic acid solution in 20 mM phosphate buffer, pH = 7.6, containing 2% methyl-β-cyclodextrin (MβCD) to assist fatty acid solubility into the isothermal titration calorimetry (ITC) cell containing As-p18 (11.68 μM) in the identical (i.e. also containing MβCD) buffer. No heats of dilution were observed in the absence of the protein. Titration data from two repetitions were analysed using a single-set-of-sites equilibrium binding model (MicroCal Origin) to give the apparent binding stoichiometry (*N*), association/dissociation constants (*K*_A_ = 1/*K*_D_) and enthalpy of binding (Δ*H*^0^).

### Preparation of NMR samples

For apo-As-p18 samples, the uniformly ^15^N- or ^13^C^15^N-enriched purified proteins, delipidated by RP-HPLC, were concentrated using Vivaspin concentrators (Sartorius) to approximately 0.5 mM in 20 mM sodium phosphate pH 7.20, and D_2_O was added to a final concentration of 5% (v/v). For holo-As-p18 with bound oleate, [U-^13^C/^15^N] As-p18 samples were incubated with natural abundance sodium oleate in a ∼4:1 (lipid:protein) ratio to ensure saturation. For NMR studies of intermolecular protein–ligand interactions, holo-As-p18 samples were prepared from [U-^13^C/^15^N] labelled protein with alternately labelled (2,4,6,8,10,12,14,16,18-^13^C_9_) sodium oleate, or with unlabelled protein with [U-^13^C] or alternately labelled sodium oleate (Sigma-Aldrich). For H/D exchange studies the As-p18/oleate complex was lyophilized and then dissolved in 99% D_2_O. The sample used for residual dipolar coupling measurements was prepared by partial alignment of As-p18 in a mechanically strained polyacrylamide gel [33,34] using a 5% non-charged acrylamide plug, cast with a 6 mm diameter and transferred into the end of a 5 mm outer diameter NMR tube (Wilmad) using a gel press and end plugs (NewEra).

### Protein X-ray crystallography

Purified protein in Tris buffer was crystallised in a solution prepared using 40% ethylene glycol, 0.1 M acetate pH 4.5, and data were collected at the Australian Synchrotron (Beamline MX2) to a resolution of 2.3 Å as described previously [23]. The structure of As-p18 was solved by molecular replacement with adipocyte lipid binding protein (34% sequence identity; ALBP; 1G74 [35]) as the search model using PHASER [36]. The structure was refined with REFMAC5 [37] and BUSTER [38] using TLS parameterisation, and inspected visually using COOT [39]. Waters, ethylene glycol and Tris were modelled into electron density in COOT before refinement. All dictionaries were obtained from the GRADE server (http://grade.globalphasing.org). Electron density was observed in the region of the proposed fatty acid binding site in each of the two chains. This was modelled as vaccenate, the predominant lipid co-purified from the bacterial expression system (see below).

The relevant statistics are presented in Table 1.

**Table 1:** Refinement statistics for crystal structure

Space group	*I*4
Unit cell (Å)	*a = b =* 102.635 *c* = 103.392
Resolution (Å) (highest resolution bin)	20.06–2.30 (2.42–2.30)
Protein residues (atoms) chain A / B	148 (1254) / 143 (1201)
Water molecules	147
Stearic acid / Tris residues	1 / 2
*R*_work_ (%)	18.57
*R*_free_ (%)	23.15
R.m.s.d. bond lengths (Å) / angles (°)	0.01 / 1.09
Average isotropic thermal parameters (Å)	
Main chain (side chain) atoms Chain A/B	41.24 (48.52) / 42.00 (48.46)
Water molecules	50.60
Stearic acid / Tris	50.74 / 58.84
Ramachandran analysis	
% Favoured regions	99.3
% Outliers	0

### Protein NMR spectroscopy and analysis

All spectra were recorded at 298 K on a 600 MHz Bruker AVANCE spectrometer at equipped with a TCI cryoprobe. The NMR experiments used and extent of assignments of the protein have been described previously [25]. NMR spectra were processed using AZARA (http://www.bio.cam.ac.uk/azara) and analysed with CCPNmr analysis [40]. Maximum entropy reconstruction [41] was used to enhance resolution of the indirect dimensions of 3D experiments. The resonance assignments were submitted to the BioMagResBank under accession number 18632. ^1^D_NH_ couplings were measured from in- phase/antiphase ^15^N-HSQC spectra [42] recorded under isotropic and partially aligned conditions***.*** Partial identification of the ligand resonances and its NOEs to As-p18 (Supplementary Figure S7) was achieved by employing 2D F1, F2-filtered NOESY, F1-filtered NOESY and F2-filtered NOESY experiments [43–45]. However, given the multiple chemical shift degeneracies of the CH_2_ groups and the limited information obtained from these experiments with natural abundance oleate, further experiments employing fully or alternately ^13^C-labelled ligand were performed in order to thoroughly identify oleate resonances and their NOEs to As-p18. To this end, ^13^C-HSQC, 2D ^13^C-NOESY-HSQC projections, 3D-HcCH-COSY and 3D ^13^C-NOESY-HSQC were particularly useful. Distance restraints for structure calculations were derived from 100 ms mixing time 3D ^15^N-NOESY-HSQC, 3D ^13^C-NOESY-HSQC spectra [46,47] recorded using labelled protein complexed with natural abundance ligand and vice versa and from 120 ms mixing time X-filtered NOESY experiments.

### Structure calculations from NMR-based restraints

A combined total of 4529 NOE-derived distance restraints were generated from the 3-D ^13^C- and ^15^N- resolved NOESY spectra, in addition to the 2D filtered and 3D experiments designed specifically to assign resonances of oleate (Supplementary Table S2). The normalized intensities were initially mapped to empirically determined upper distance limits and the distances recalibrated during the calculations by ARIA 2.3/CNS [48,49]. Ambiguous restraints were included at all stages of the calculation and disambiguated to a contribution level of 0.9 in the final iterations. 236 φ or ψ backbone dihedral angle constraints generated using DANGLE [50] were applied as flat bottomed harmonic restraints with wide (±15–35°) bounds only during the high temperature phase of the calculations. Topology and parameter definitions for the oleate molecule were incorporated into the PARALLHDG5.3 forcefield using XPLO2D [51] with PDB entry 2flj as a template [52] and incorporating parameters for the vinyl atoms [53]. The ARIA ions.link and atomnames.xml files were modified to allow recognition of the ligand. Hydrogen bond restraints (1.7 Å < d_H,O_ < 2.2 Å and 2.7 < d_N,O_ < 3.2 Å) were included during later rounds of structure calculation for amide protons whose signals were still observed in a ^15^N-HSQC spectrum recorded 18 h after re-dissolution of a lyophilized sample in D_2_O. 54 hydrogen-bond acceptors were identified by inspection of the NOE-refined structures where they were also supported by NOE data.

Restraints derived from 113 ^1^D_NH_ RDCs were incorporated by estimating the average alignment tensor using the NOE+H-bond ensemble structures and the measured RDCs in PALES [54]. This alignment tensor was then used to apply the RDC restraints using the SANI potential [55] in square-well mode.

The final ensemble of structures comprises the 20 lowest restraint energy structures from 100 generated in the final iteration. These structures were refined in explicit water [56] and superposed and the structure closest to the mean of the ensemble was chosen as representative.

## Results

### The nemFABP family

**Figure 1:**
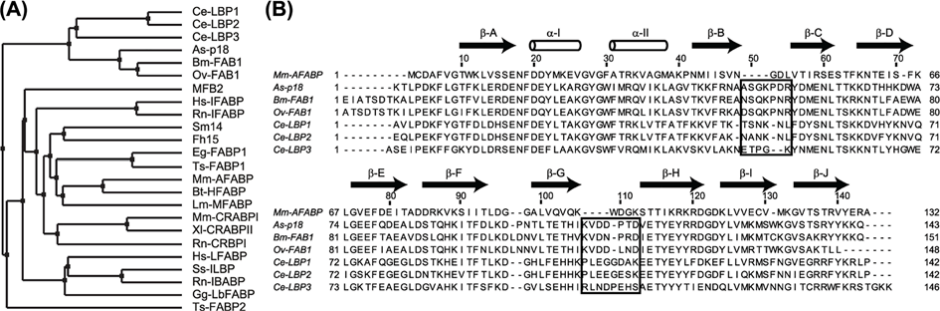
Unique features of the nemFABP sub-family (**A**) Average distance dendrogram of a MUSCLE multiple sequences alignment of representative FABPs from a diverse set of organisms. As-p18 clusters with nemFABP sequences from other nematodes, here represented by *Caenorhabditis elegans* (Ce) and *Brugia malayi* (Bm). The FABPs retrieved from the NCBI protein database were: MFB2 (P31417), Hs-LFABP (P07148), Ss-ILBP (P10289), Gg-LbFABP (P80226), Rn-IBABP (P80020), Hs-IFABP (P12104), Rn-IFABP (P02693), Rn-CRBPI (P02696), Mm- CRABPI (P62965), Xl-CRABPII (P50568.2), Ts-FABP2 (ADZ72849.1), Eg-FABP1 (Q02970), Ts- FABP1 (ADZ72848.1), Lm-MFABP (P41509), Mm-AFABP (P04117), Bt-HFABP (P10790), Sm14 (P29498), Fh15 (Q7M4G0.3), Ce-LBP1 (Q20223), Ce-LBP2 (Q20224), Ce-LBP3 (Q27GU2), As-p18 (AAA98565.1), Bm-FAB1 (AF178439), Ov-FAB1 (BF727540). (**B**) Structure-based sequence alignment of As-p18 and the closest FABP with known structure, Mm-AFABP, with other nemFABP sequences included. The sequence elements that are consistently extended in the nemFABP subfamily are boxed. Arrows and cylinders indicate β-strands and α-helices in As- p18.

Nematodes produce proteins that are members of the FABP/iLBPs family, which cluster with those of vertebrates and other metazoan groups [22,57,58], but the nemFABPs form a separate subfamily (Figure 1A), and in addition, are the only FABPs expressed with N- terminal secretion signal peptides other than in tardigrades. Figure 1B compares the amino acid sequence of As-p18 with those of other nemFABPs from nematodes and with the most similar mammalian FABPs of known structure, adipocyte FABP from mouse (Mm-AFABP), illustrating the sequence features that are exclusive to the nematode sequences. While FABP sequence length generally varies little around 130 amino acids, nemFABPs are usually 10–16 residues longer, predominantly due to insertions that appear from the alignment to be situated in the loops between strands βB and βC and near the loop between strands βG and βH, assuming a canonical FABP structure.

### Recombinant As-p18 co-purifies with fatty acids

**Figure 2:**
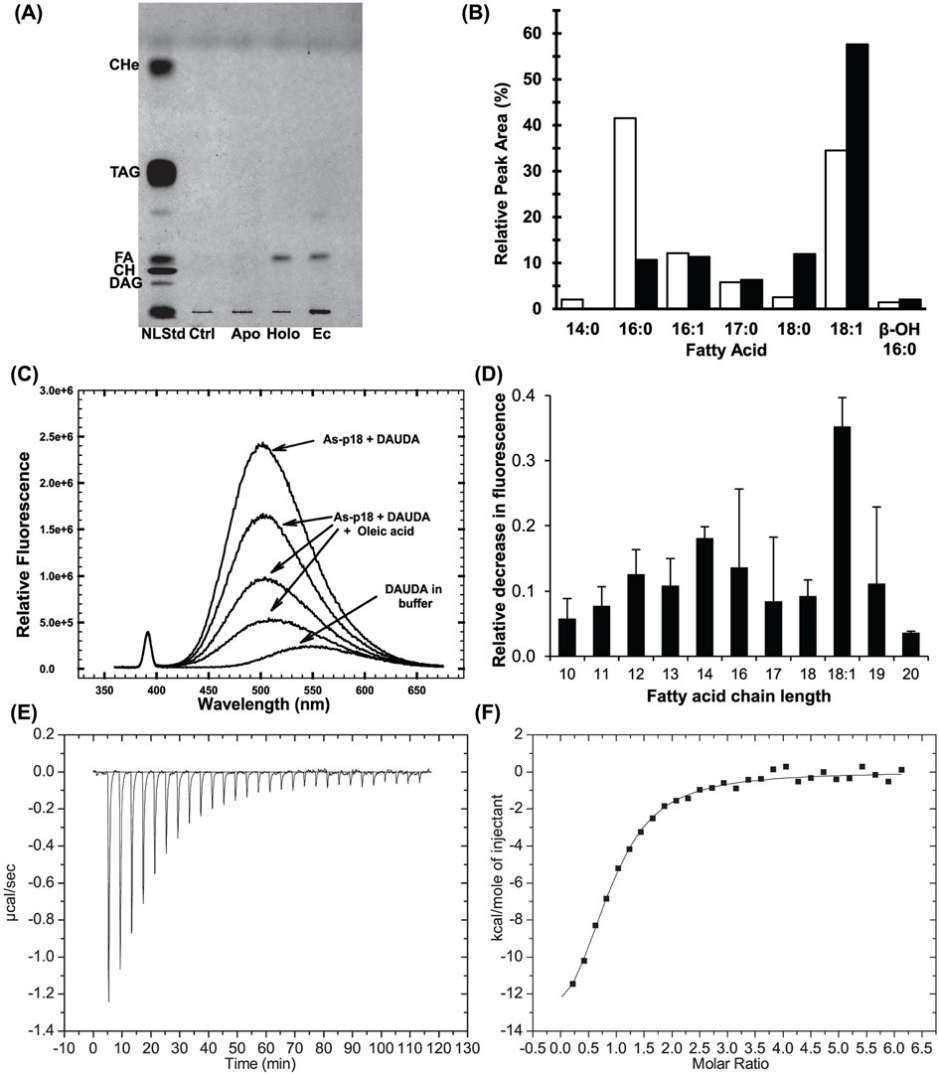
Ligand binding by As-p18 (**A**) Thin layer chromatography of the lipids that co-purify with As-p18 under conditions that resolve fatty acids and neutral lipids. Holo and Apo, As-p18 purified without or with an HPLC step; NLStd, neutral lipid standard mixture from rat liver homogenate; Ctrl, buffer and solvents only; Ec, *E. coli* extract; CH, cholesterol; FA, free fatty acid; TAG, triacylglycerols; CHe, cholesterol ester. (**B**) Relative peak areas of the GC peaks of the methyl esters of the fatty acids that co-purify with As-p18 (filled bars) or that are present in *E. coli* extract (unfilled bars). (**C**) Fluorescence emission spectra (excitation at 345 nm) of 1 μM DAUDA in buffer or on the addition of 1.25 μM rAs-p18 and following the competitive displacement of DAUDA from rAs-p18 by the progressive addition of oleic acid: three additions of 10 μl of 1:100 dilution of a 10 mM stock solution of oleic acid were added directly to the fluorescence cuvette. (**D**) The relative decrease in fluorescence emission at 500 nm of the fatty acid analogue, DAUDA, upon displacement from As-p18 by fatty acids reveals the efficacy of 0.49 μM competing saturated or unsaturated (18:1) fatty acids. (**E**) Baseline corrected ITC data for the injection of 2% methyl-β-cyclodextrin-solubilized oleate into a solution of As-p18, and (**F**) the data plotted to show enthalpy per mole of oleate injected versus molar ratio and the fitted binding isotherm.

Lipid-binding proteins often co-purify with *E. coli*-derived ligands when produced recombinantly [59–61]. TLC showed that recombinant As-p18 only co-purified with fatty acids (Figure 2A). In contrast to several other types of lipid-binding proteins, no other classes of lipid were present in the purified As-p18 extract. Fatty acids in *E. coli* include saturated, monounsaturated, β-hydroxy and cyclopropane species, which have chain length of C12 to C18 [31]. Linolenic acid (18:3) has also been reported to be synthesized by *E. coli* [62]. The relative prevalence of these ligands varies according to growth temperature, growth rate and the fatty acid content of growth media [31,62,63]. As a first approach to assessing ligand preference in a cellular environment, lipids extracted from purified As-p18 or the originating bacterial culture were analysed. *E. coli*’s cytoplasm is unlikely to be directly analogous to the natural environment of As-p18, but analysis of the bacterial lipids bound should contribute to an understanding of the protein’s ligand-binding repertoire. GC analysis of the FAMEs prepared from *E. coli* extract or from purified As-p18 revealed enrichment in 18-carbon fatty acids. In *E. coli*, these are principally 18:1 and for As-p18 almost exclusively vaccenate (cis-11-octadecenoate). Enrichment for stearate was also detected, although to a lesser extent (Figure 2B).

### Ligand binding by As-p18

In order to gain further insight into As-p18’s ligand preferences, fluorescence-based ligand-binding experiments were performed. As-p18 has previously been shown to bind a fluorescently tagged fatty acid (DAUDA) that is displaceable by a natural fatty acid (oleic; Figure 2C), but not retinol [15]. The preference of As-p18 for fatty acids of different chain lengths was investigated here by the addition of equimolar amounts of saturated fatty acids with different chain lengths (ranging from C_10:0_ to C_20:0_) and unsaturated C_18:1_ to a pre-formed complex of As-p18:DAUDA (Figure 2D). Displacement of DAUDA is readily observed as a reduction in fluorescence emission intensity and a red-shift in DAUDA emission spectrum of the mixture (Figure 2C), which proved to be maximal for the monounsaturated fatty acid, oleic. A similar bias has also been indicated for the As-p18 homologue in the filarial parasitic nematode *B. malayi* [17].

Oleic acid was chosen as a suitable ligand to characterize fatty acid binding to As-p18 by ITC (Figure 2E,F). Satisfactory binding curves, consistent with a 1:1 binding stoichiometry (*N* = 0.90 ± 0.05), were obtained. The fitted dissociation constant (*K*_D_) of 3.1 ± 0.2 × 10^−6^ M is comparable to the *K*_D_ exhibited by A-FABP for the same ligand [64]. The enthalpic contribution was determined to be the driving force for the binding event (−15 ± 1 kcal/mol), compensating for the unfavorable entropic contribution to Δ*G* (−*T*Δ*S* = 6.9 ± 0.9 kcal/mol). The large enthalpic contribution in fatty acid binding is typical for the FABP family and it is mainly attributed to stabilization of the carboxylate inside the protein binding pocket through polar interactions [64,65].

### As-p18 exhibits a canonical FABP fold but with features exclusive to nemFABPs

We were able to determine the 3D structure of As-p18 by X-ray crystallography (Table 1) and solution NMR spectroscopy (Table 2). Using a micro-focus beamline, a dataset was collected with diffraction observed to beyond 2.2 Å. Due to clear evidence of radiation damage, the data were processed and scaled to a maximum resolution of 2.3 Å [23]. As-p18 crystalized with two near-identical copies of the protein in the asymmetric unit (coordinate RMSD 0.19 Å for all equivalent heavy atoms), but with incomplete electron density in the ligand-binding pockets. For the solution structure, NMR data from a combination of As-p18 samples with different labelling patterns including ^15^N,^13^C labelled protein with unlabelled oleate, and unlabelled protein with uniformly or alternately ^13^C labelled oleate, were used to derive the distance and chemical shift restraints used to calculate the structures. The NMR structures are well defined with backbone root-mean-square deviation (RMSD) for residues 1–143 of 0.53 Å. When only the regions of regular secondary structure are included the RMSD falls to 0.41 Å.

**Table 2:** Experimental restraints and statistics of NMR structures

NOE distance restraints	
Total NOE restraints	4529
Ambiguous	1687
Unambiguous	2842
Intra-residue	1395
Inter-residue	1447
Sequential (|*i-j*| = 1)	636
Medium-range (1 < |*i-j*| < 5)	225
Long-range (|*i-j*| ≥ 5)	586
Violations per structure > 0.5 Å	0.05
Violations per structure > 0.3 Å	2.90
Distance restraint RMSD	0.023Å
Other restraints	
RDCs ^1^D_NH_ restraints	113
Q factor	0.33
Hydrogen bond restraints	108
DANGLE dihedral angle restraints	236
Coordinate RMSD (Å)^2^	
Backbone heavy atoms^1^	0.88
All heavy atoms^1^	0.53
Parameter RMSD from idealised geometry	(mean and SD)
Bond lengths (Å)^2^	0.01 ± 0.00
Bond angles (°)	0.57 ± 0.02
Impropers (°)	1.67 ± 0.06
Ramachandran statistics^1^ (%)	
Most favoured	82.2
Additionally allowed	16.3
Generously allowed	0.8
Disallowed	0.7

^1^Residues 1–143

^2^To the unbiased mean structure

**Figure 3:**
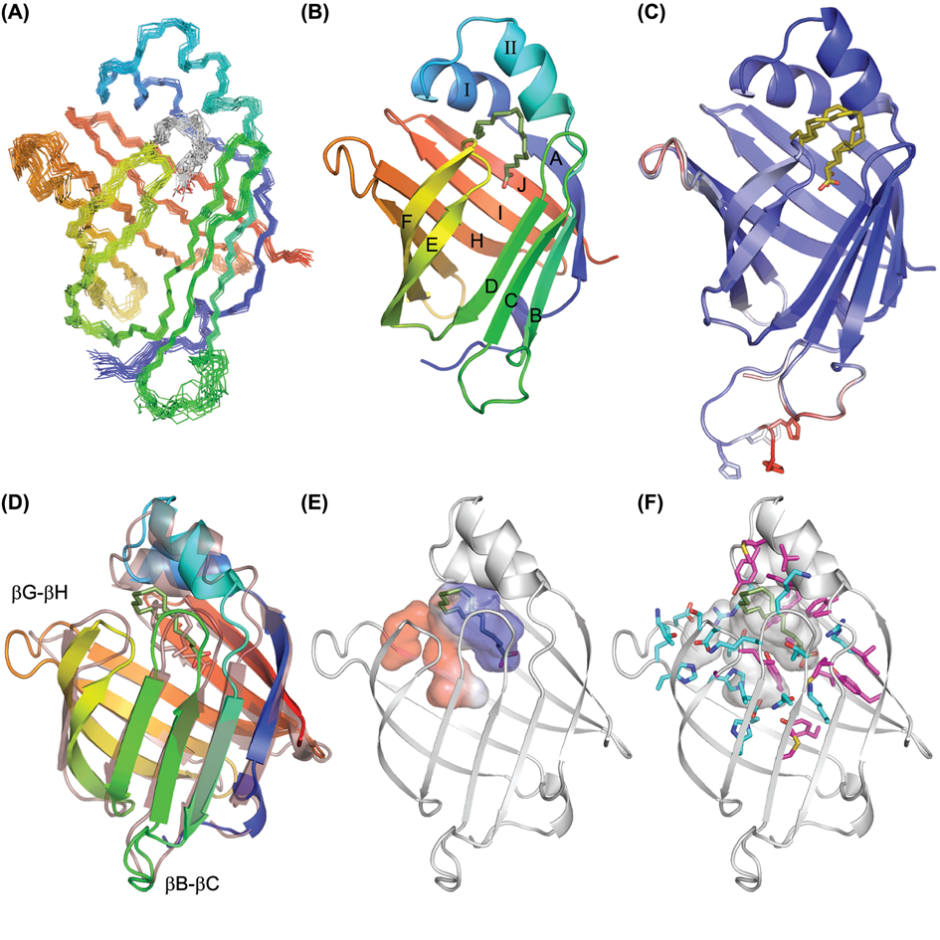
The 3D structure of As-p18 (**A**) The ensemble of the 20 lowest energy (from a total of 100) NMR structures refined in explicit solvent superimposed on the backbone heavy atoms (PDB: 6I9F). The backbone nitrogen and carbon atoms are shown and the molecule is coloured from blue (N-terminus) to red (C-terminus). Oleate is shown as grey and red sticks. (**B**) Cartoon representation of the representative NMR structure with the regular secondary structure elements labelled and oleate depicted in stick form and coloured olive green. (**C**) The two X-ray structures of As-p18 in complex with vaccenate (brown sticks; PDB: 6I8X) that are found in the asymmetric unit superimposed and shown in cartoon form and coloured according to B-factor (from 27 Å^2^ (blue) to 81 Å^2^ (red)). The ordered His-tag residues of one copy of As-p18 in the asymmetric unit are shown with the sidechains as sticks. (**D**) Comparison of the superimposed A-FABP (transparent; PDB: 1HMS) and As-p18 highlighting the positions of the extended nemFABP loops. (**E**) The ligand binding cavity is shown in surface representation and coloured by electrostatic surface potential (red, negative; blue, positive) within the backbone cartoon. (**F**) Arrangement of the hydrophobic (magenta sticks) and hydrophilic (cyan sticks) side-chains that line the interior of the cavity As-p18. (D), (E) and (F) are rotated 45° about a vertical axis compared to (A), (B) and (C).

The NMR and X-ray-derived structures of As-p18 agree closely (RMSD = 0.46 Å) and exhibit features that characterize the FABP family [1], namely the β-clamshell structure comprising 10 antiparallel β-strands capped by a helix-turn-helix section. As in other FABPs, there is a defect between strands βD and βE of the barrel where no inter-strand main chain hydrogen bonds are apparent (corroborated by D_2_O/H_2_O exchange experiments (Supplementary Figure S2)). The central cavity is lined with polar and hydrophobic amino acids side-chains present in approximately even proportions, but unevenly distributed, and a ‘portal’, postulated to be the entrance for ligand molecules, is delimitated by the turns βC-βD and βE-βF and α-helix II. In both the NMR- and X-ray-derived structures, the N-terminal residues Asp5-Phe7 form a 3_10_- helical loop that is an extra secondary structure element characteristic of the subfamily IV in mammalian FABPs [52]. Out of the 43 side chains that project into the protein’s internal cavity, 23 are hydrophobic (Ala, Val, Ile, Leu, Phe or Met), 20 are hydrophilic (Arg, Lys, His, Glu, Asn, Asp or Thr) and 5 are tyrosines (which have both hydrophobic and hydrophilic character). As shown in Figure 3, these residues are arranged in three zones: the bottom of the cavity with all hydrophobic side-chains; a middle section enriched in hydrophilic and ionizable groups; and a third region in the vicinity of the portal exhibiting a mixture of residue types that interact with the ligand (see below). This arrangement resembles that found for heart-FABP (H-FABP; FABP3), where the binding cavity is also divided into three sections [66]. The solvent-accessible volume of the ligand-binding cavity in the solution structure of As-p18 complexed with oleate calculated using the program CASTp [67] is 785 Å^3^, using a probe radius of 1.4 Å. Within the cavity there are exchangeable side chain protons that exhibit reduced rates of exchange with solvent, indicative of involvement in hydrogen-bond networks, another common characteristic of FABPs [68]. The resonances concerned are His69N_δ1_H_δ1_, Thr62O_γ1_H, Tyr118OH and Thr136O_γ1_H, observed at 169.1/13.45 ppm; 6.74 ppm; 10.021 and 4.39 ppm, respectively, which were identified by their crosspeaks to neighbouring hydrogens in the same residue in X-filtered NOESY spectra recorded following lyophilization of the sample and dissolution in D_2_O.

Notwithstanding its canonical FABP fold, As-p18 exhibits features that differentiate it from any FABP structure previously determined. The loop between strands βB and βC is twice as long as that of mouse adipocyte FABP (A-FABP; FABP4), which is the most similar non-nematode FABP to As-p18 [15] (Figure 3D). Where mouse A-FABP has 4 residues (Asn45, Gly46, Asp47, Leu48), As-p18’s loop harbours 8 residues (Ala48, Ala49, Ser50, Gly51, Lys52, Pro53, Asp54, Arg55) with charged sidechains that invest it with an overall net positive charge instead of a negative one as in A-FABP. The βB-βC loop is poorly defined in solution, consistent with the high crystallographic B factor values for this region, but there is limited evidence for flexibility from the ^15^N relaxation data (Supplementary Figure S1). The loop between strands βG and βH (the so-called ‘Ω loop’; [57]) is also extended in As-p18, containing six residues (Val107, Asp108, Asp109, Pro110, Thr111, Asp112) and carrying a net negative charge whilst in mouse A-FABP this loop of only three residues (Asp98, Gly99, Lys100) has a net neutral charge. Currently available empirically-derived iLBP structures indicate that these extended loops exist only in nemFABPs (Figure 1). Sequence alignments and theoretical tertiary structure modelling have led to the suggestion that similar extended loops may occur in an FABP from a cestode parasite [69], but, as found with As-p18, sequence alignments of FABPs and homology modelling can be misleading as to the length and position of these loops.

As-p18 also displays protruding hydrophobic residues flanking the portal region, the most prominent being Trp30, Ileu31, Val35 and Leu38. These conserved bulky hydrophobic side chains have been dubbed ‘sticky fingers’ [70], and are potentially involved in protein–membrane or protein–protein interactions, or to facilitate capture of hydrophobic ligands. Perhaps not coincidentally, such sticky fingers are present in FABPs which exchange ligands by collisional contact with membranes [71–73], but not in one FABP that does not carry out ligand exchange in this way [16,59]. Trp30 and Leu38 of As-p18, are positioned and solvent-exposed similarly to Phe27 and Met35 on the α-helix II of mouse A-FABP, respectively.

### Ligand–protein interaction

In the crystal structure of As-p18, the electron density associated with the co-purifying ligand was of insufficient resolution to determine its conformation unambiguously. The electron density within the binding pocket [74] indicates that a single resident fatty acid molecule could be in a U-shaped conformation as observed for other FABPs [64,75–79], and sit with its charged head group either buried within the protein’s binding pocket, or pointing out towards the pocket’s opening (Supplementary Figure S8). The head group buried orientation is the more likely given the precedence set by other FABPs and our NMR data (Figure 3A). Since As- p18 predominantly co-purifies with vaccenate (Figure 1A,B and Supplementary Figure S3), we fitted this ligand into both copies of the protein in the asymmetric unit (Figure 3C).

**Figure 4:**
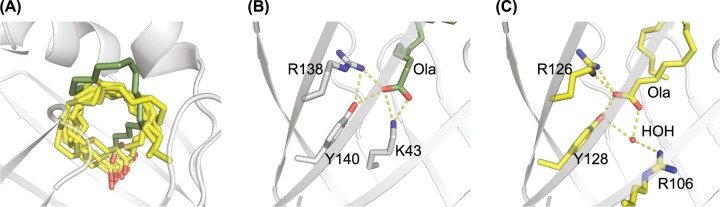
Details of ligand binding by As-p18 (**A**) Comparison of the positions adopted by various ligands (in yellow) in the cavities of FABPs: Arachidonate in A-FABP (PDB: 1ADL); oleate in HFABP and SjFABP (PDB: 1HMS and 1VYF, respectively); palmitate in EFABP and EgFABP (PDB: 1B56 and 1O8V, respectively). The backbone cartoon of As-p18 is shown with the bound oleate shown in olive green. Comparison of the ligand carboxylate recognition sites in As-p18 (**B**) and A-FABP (**C**) with key residues shown in stick form and labelled with their residue number. Hydrogen bonds are shown as dashed yellow lines.

Significant NMR chemical shift differences were observed between apo-As-p18 and oleate-saturated forms of the protein and 17 amide resonances that were not seen in the apo- form became visible in the NMR spectra (Supplementary Figures S4,S5 and S6). The chemical shift changes were most significant in the strands and loops surrounding the ligand binding site, and the residues that became visible upon ligand binding are located in the capping helices and the βC-βD loop (Supplementary Figure S6). We calculated the solution structure of As-p18 that had been stripped of ligand and saturated with oleate as a complex with a single molecule of oleate, the stoichiometry indicated by our ITC (Figure 2E,F) and NMR titration data. Dictated by the 16 unambiguous NOE-derived intermolecular distance restraints (Supplementary Table S1 and Supplementary Figure S7) and additional ambiguous restraints, the NMR structure clearly reveals the position and orientation of the oleate molecule, occupying the upper part of the protein’s cavity close to the portal (Figure 3A,B), as is typical of subfamily IV iLBPs [68]. Its overall conformation is similar to the slightly twisted U-shaped conformation of fatty acids in the holo-forms of A-FABP, muscle-FABP, epidermal-FABP, and brain-FABP (1ADL, 1HMS, 1B56, 1FDQ, respectively) [76,77,79,80] but the position of the oleate’s carboxylate is less deeply buried than in the mammalian counterparts. The way in which the fatty acid curls inside the binding pocket is also unusual. From C1 to C10, the carbon chain extends towards the loop between βC and βD and then turns to place C11-14 between the βΕ-βF loop and αII before diving back into the cavity placing the terminal methyl group between αI and αII (Figure 3B). In the holo-FABP forms from mammals, their ligands describe a similar path, albeit in the opposite direction, with the end of the hydrocarbon tail located against residues on strands βC and βD (Figure 4).

The carboxylate group of oleate is buried towards the bottom of As-p18’s cavity, in close proximity to the hydroxyl group of Tyr140, the guanidinium group of Arg138 and the ε-amino group of Lys43. Figure 4 shows the interactions of the fatty acid carboxylate with the trio of side chains. Tyr140 and Arg138 match two of the carboxylate tethering triad of residues found in other FABPs [81], as seen in group IV FABPs [82,83] and invertebrate FABPs [52,75,78,84]. The nemFABPs’ network, as exemplified by As-p18, is composed differently, with a conserved lysine (Lys43) from strand βB replacing the third, water-mediated interaction with an arginine at the C-terminal end of strand βH. The strand βH arginine (Arg106 of A-FABP) is replaced by at tyrosine in nemFABPs, and this residue is not directly involved in ligand binding.

## Discussion

We report the first structure of a member of the nematode-specific nemFABPs, which are highly unusual within the FABP/CRBP/CRABP family of iLBPs of metazoans in that they possess a secretion signal peptide, are secreted from the synthesizing cell, and have structural modifications that have not been seen before. In other respects the protein’s fold is typical of an iLBP, as is the position and nature of its binding site for hydrophobic ligands, and the orientation and tethering of ligand in the binding site. The unusual features include two extended loops, one immediately adjacent to the portal through which ligands are thought to enter and leave the binding pocket of iLBPs, and another on the opposite side of the molecule from the portal, and the triad of amino acid side chains that tether bound fatty acids is differently arranged and composed.

The larger of the two unusual loops links strands βB and βC, and none as large as this has yet been observed in any iLBP structure obtained by crystallography or NMR. As this loop is distant from the portal, it seems unlikely to be directly involved in ligand acquisition and release, and could instead be involved in interaction with other proteins, either in solution or membrane-bound. Its mix of amino acids with charged and apolar characteristics would seem to argue against its involvement in interaction with plasma membranes through hydrophobic interactions, though interaction with a membrane surface by other mechanisms remains possible. The smaller extended loop in As-p18 links strands βG and βH, immediately adjacent to the portal and also displays no particular bias in its amino acid composition. This loop, classified in FABPs as an ‘Ω loop’ [57,85], varies in length and disposition amongst iLBPs, though that of As-p18 is unusually long.

FABPs can be divided into those that exchange ligands with membranes either by direct, collisional contact, during which the ligand need not enter the aqueous phase, and those in which exchange instead involves transient ligand entry into solvent [2,86]. If As-p18 interacts by direct contact with a membrane or other structure in the acquisition or delivery of lipids, then this βG-βH loop is in a strategic position to be involved. In those FABPs that appear to interact by collisional mechanism, there is abundant evidence that a crucial interaction site lies on the two α-helices adjacent to the portal [87–90], in particular Lys side chains on helix αI [2,86]. Moreover, all FABPs that are known to operate in this way carry a signature group of solvent-exposed large apolar amino acids immediately beside the portal region [70]. As-p18 also possesses such a ‘sticky finger’ that comprises three consecutive apolar side chains (Trp30, Ile31 and Met32) on helix αII, and a near neighbour (Val35), all of which project into the solvent. This remains an untested association, but the role of such an unusually placed group of hydrophobic amino acids deserves an explanation.

As-p18 and its homologues in other nematodes, both parasitic and free-living, are secreted into the perivitelline space surrounding developing embryos within the egg. Possible functions of As-p18 include interaction with cell surface receptors in the recovery of lipids from perivitelline fluid that have been lost from the embryo, or those deposited in the egg prior to shell formation. It may also be that the protein is involved in regulating the concentration of lipids in the fluid or removing damaged forms. Nematode eggs can be remarkably environmentally robust [91,92]; *Ascaris* eggs, for instance, can survive viable in soil for almost a decade [93,94]. Such environmental resilience would likely require maintenance of a lipid layer of the eggshell, which is likely to suffer from oxidative degradation with time, requiring removal of damaged lipids and their replacement. The main lipids in *Ascaris* eggshells are ascarosides, and it is yet unknown whether As-p18 binds them or their degradation products. In the case of the filarial nematode parasite *B. malayi*, the eggs are retained within the female worm until hatching, so maintenance of a robust eggshell lipid layer may not seem to be as critical. The nemFABP in this parasite’s perivitelline fluid may therefore be more involved in regulating lipid balance of the fluid or acquiring and delivering to the embryos maternally-produced small lipids that percolate through the shell.

NemFABPs have structural characteristics and perform a biological function that has not been investigated before. Whether any of the unusual features of these proteins relate to their function within nematode eggs remains to be defined, but means are now available (such as *in vivo* genetic modification of perivitelline nemFABP of *C. elegans*) to tackle such questions and to find out what it is about nematodes that requires such unusual relatives of the iLBPs.

## Supporting information

**Supplementary Figure S1:** As-p18 ^15^N relaxation parameters.

**Supplementary Figure S2:** Amide proton hydrogen-deuterium exchange in oleate-bound As-p18 monitored by NMR spectroscopy.

**Supplementary Figure S3:** Identification of As-p18’s co-purifying ligand by GC-MS.

**Supplementary Figure S4:** Overlaid ^15^N HSQC NMR spectra of As-p18 with and without oleate.

**Supplementary Figure S5:** Histogram of the chemical shift perturbations induced in the backbone amides of As-p18 by oleate binding.

**Supplementary Figure S6:** Positions of amino acids in As-p18 that undergo the greatest chemical shift perturbations between *apo* and *holo* forms.

**Supplementary Figure S7:** Intermolecular NOEs observed in samples of unlabelled As-p18 loaded with with [U-^13^C] or alternately labelled sodium oleate.

**Supplementary Figure S8:** Electron density in the ligand binding pocket of As-p18.

**Supplementary Table S1:** ^1^H chemical shift coordinates and resonance assignments for the intermolecular NOEs observed between As-p18 and bound oleate

**Supplementary Table S2:** Chemical shift assignments for oleic acid bound to As-p18

## Abbreviations

CRABP: cellular retinoic acid-binding protein
CRBP: cellular retinol-binding protein
DAUDA: 11-[(5-dimethyl-aminonaphalene-1-sulfonyl)amino]undecanoic acid
FA: fatty acid
FAME: fatty acid methyl ester
GC: gas chromatography
HSQC: heteronuclear single-quantum correlation spectroscopy
iLBP: intracellular lipid-binding protein of which FABPs, CRBPs and CRABPs are members
ITC: isothermal titration calorimetry
nemFABP: nematode fatty acid-binding protein
NMR: nuclear magnetic resonance
NOE: nuclear Overhauser effect
NOESY: nuclear Overhauser and exchange spectroscopy
OLA: oleic acid/oleate
RDC: residual dipolar coupling
RMSD: root-mean-square deviation
rpm: revolutions per minute
TLC: thin layer chromatography
