# Supplementary material for: Structure and ligand binding of As-p18, an extracellular fatty acid binding protein from the eggs of a parasitic nematode
Source: Biosci Rep. 2019 Jul 23;39(7):BSR20191292. doi: 10.1042/BSR20191292 (PMC6646235; doi:10.1042/BSR20191292)
Supplement: Supplementary file 1 [file bsr20191292_Supp1.pdf]

Asp-18, an extracellular fatty acid binding protein of nematodes, exhibits unusual structural features

Marina IBÁÑEZ-SHIMABUKURO*, M. Florencia REY-BURUSCO*, Mads GABRIELSEN†, Gisela R. FRANCHINI*, Alan RIBOLDI-TUNNICLIFFE‡, Andrew J.ROE†, Kate GRIFFITHS§, Alan COOPER‖, Betina CÓRSICO*, Malcolm W. KENNEDY§¶[1] Brian O. SMITH¶[1]

*Instituto de Investigaciones Bioquímicas de La Plata, CONICET-UNLP, Facultad de Ciencias Médicas, calles 60 y 120, 1900-La Plata, Argentina, †Institute of Infection, Immunity and Inflammation, University of Glasgow, ‡Australian Synchrotron, Clayton, Victoria, Australia, §Institute of Biodiversity, Animal Health and Comparative Medicine, University of Glasgow G12 8QQ, UK, ‖School of Chemistry, University of Glasgow, Glasgow G12 8QQ, UK, ¶Institute of Molecular, Cell & Systems Biology, University of Glasgow G12 8QQ, UK

Supplementary material

Table S1. ^{1}H chemical shift coordinates and resonance assignments for the intermolecular NOEs observed between As-p18 and bound oleate

Table S2. Chemical shift assignments for oleic acid bound to As-p18

Figure S1. As-p18 ^{15}N relaxation parameters.

Figure S2. Amide proton hydrogen-deuterium exchange in oleate-bound As-p18 monitored by NMR spectroscopy.

Figure S3. Identification of As-p18's co-purifying ligand by GC-MS.

Figure S4. Overlaid ^{15}N HSQC NMR spectra of As-p18 with and without oleate.

Figure S5. Histogram of the chemical shift perturbations induced in the backbone amides of As-p18 by oleate binding.

Figure S6. Positions of amino acids in As-p18 that undergo the greatest chemical shift perturbations between *apo* and *holo* forms.

Figure S7. Intermolecular NOEs observed in samples of unlabelled As-p18 loaded with with [U-^{13}C] or alternately labelled sodium oleate.

Figure S8. Electron density in the ligand binding pocket of As-p18.

Abbreviations (or in full first mention in text)

CSP, NOE, OLA, HSQC, FAME (already given in text),

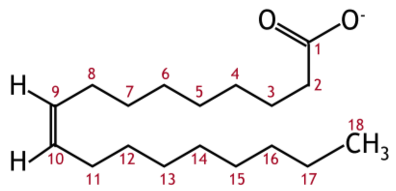

As-p18		OLA	
Residue and Atom	δ ^{1}H (ppm)	Residue and Atom	δ ^{1}H (ppm)
36 Ile $H_{\delta1*}$	-0.03	1001 H9	5.167
36 Ile H_{α}	3.43	1001 H9	5.167
36 Ile $H_{\gamma2*}$	0.83	1001 H9	5.167
32 Met $H_{\varepsilon*}$	2.00	1001 H10	5.167
36 Ile $H_{\delta1*}$	-0.03	1001 H10	5.167
32 Met $H_{\varepsilon*}$	2.00	1001 $H12_{1}$	1.152
84 Asp H_{α}	4.22	1001 $H12_{2}$	1.235
36 Ile $H_{\delta1*}$	-0.03	1001 $H16_{2}$	1.108
28 Tyr $H_{\varepsilon*}$	6.49	1001 H18*	0.832
19 Phe $H_{\varepsilon*}$	6.94	1001 H18*	0.832
36 Ile H_{α}	3.43	1001 H18*	0.832
23 Leu H_{α}	3.58	1001 H18*	0.832
36 Ile $H_{\delta1*}$	-0.03	1001 H18*	0.832
23 Leu $H_{\delta1*}$	0.44	1001 H18*	0.832
19 Phe H_{ζ}	6.72	1001 H18*	0.832
23 Leu $H_{\delta2*}$	0.14	1001 H18*	0.832

Table S1. ^{1}H chemical shift coordinates and resonance assignments for the intermolecular NOEs observed between As-p18 and bound oleate that could be assigned unambiguously.

**C1**	**C2**	**C3**	**C4**	**C5**	**C6**	**C7**	**C8**	**C9**
-	41.01	30.33	32.78	-	32.75	32.72	29.35	-
	**H2**	**H3**	**H4**	**H5**	**H6**	**H7**	**H8**	**H9**
	1.84<br>1.84	1.39<br>1.39	1.20<br>1.20	-	1.20<br>1.20	1.21<br>1.34	1.81<br>1.81	5.17

**C10**	**C11**	**C12**	**C13**	**C14**	**C15**	**C16**	**C17**	**C18**
133.52	28.86	31.26	30.96	31.73	32.45	34.28	25.44	17.36
**H10**	**H11**	**H12**	**H13**	**H14**	**H15**	**H16**	**H17**	**H18***
5.17	1.84<br>1.84	1.15<br>1.24	0.92<br>1.01	1.01<br>1.01	1.21<br>1.21	1.04<br>1.11	1.19<br>1.24	0.83

Table S2. Chemical shift assignments for oleic acid bound to As-p18

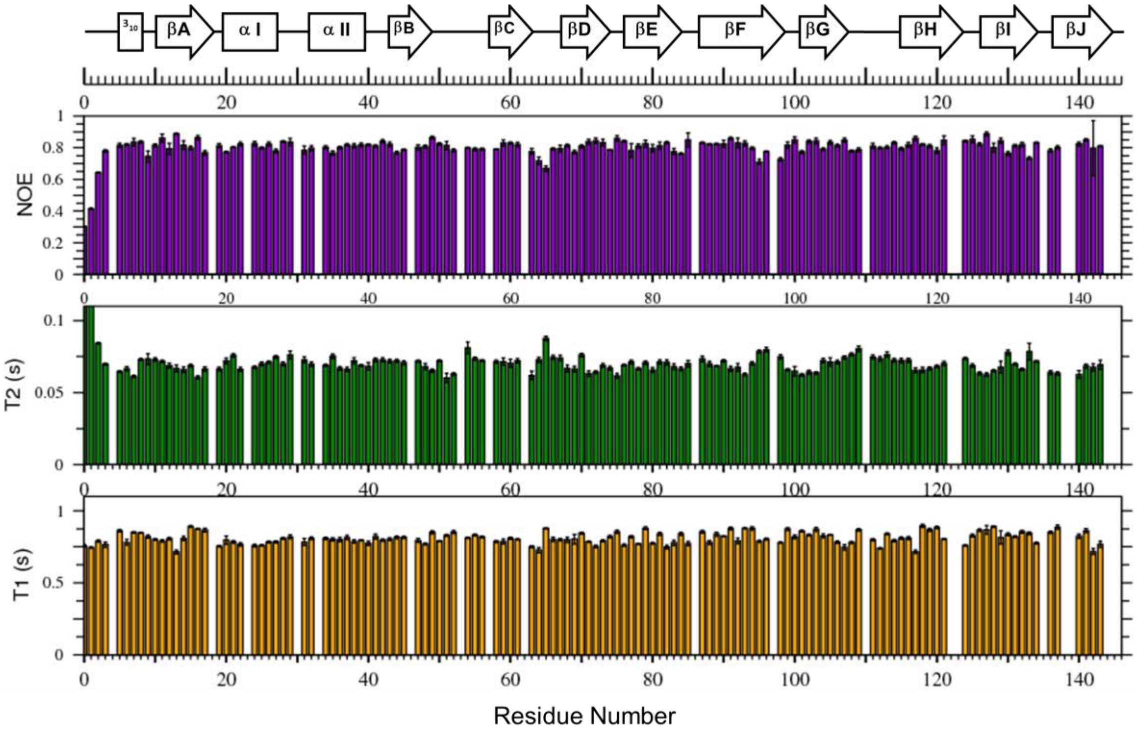

Figure S1. ^{15}N relaxation parameters for As-p18 recorded at 298 K and 14.1 T (60.8 MHz for ^{15}N). T_1, T_2 and heteronuclear NOE (I_{sat}/I_{ref}) values are plotted with their error estimates for each well resolved HSQC crosspeak. The locations of regular secondary structure elements are indicated by the cartoon above. Slight dips in heteronuclear NOE values are seen for the βC to βD and βF to βG loops, accompanied by a few residues with longer T_2 values in the middle of each loop. The T_1 values remain relatively constant throughout. This picture is consistent with increased, fast timescale dynamics for the loops.

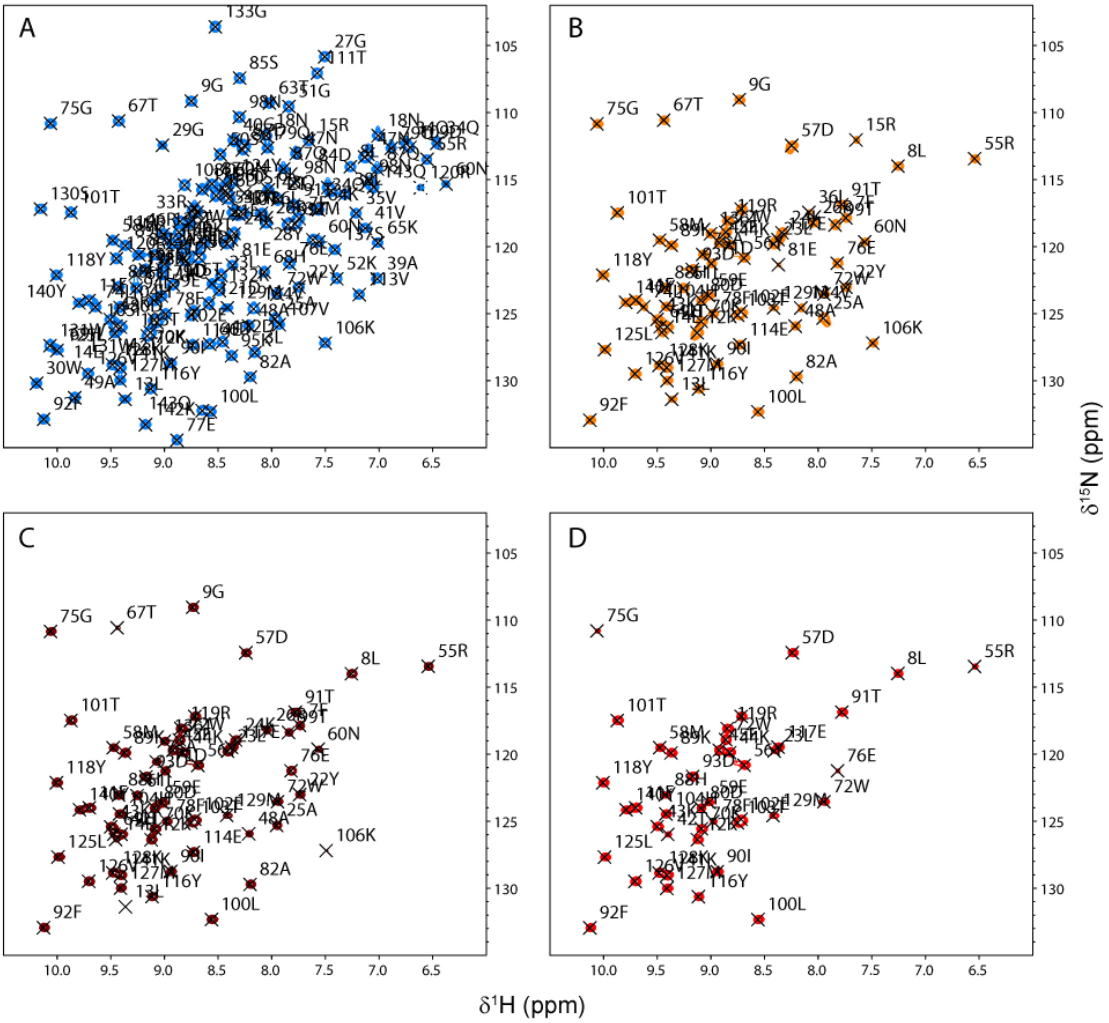

Figure S2. Amide proton hydrogen-deuterium exchange in oleate-bound As-p18 monitored by NMR spectroscopy. ^{15}N HSQCs recorded (A) before lyophilisation and (B-D) starting approximately 10 minutes, 18 minutes and 20 hours after resuspension in D_2O.

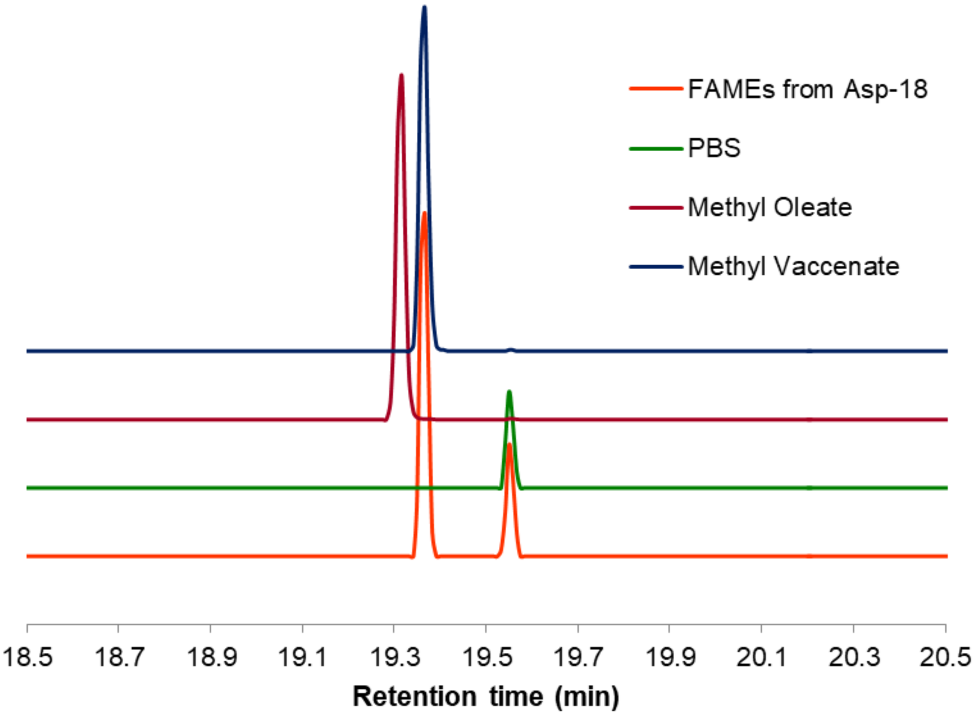

Figure S3. Identification of As-p18's co-purifying ligand by GC-MS. Overlay of GC-MS chromatograms of fatty acid methyl esters (FAMEs) prepared from As-p18 extract, from phosphate buffered saline solution (PBS) subjected to the same extraction and derivatization protocol, and FAMEs prepared from oleate and vaccenate as standards. FAMEs were injected onto a DB-35ms GC column (Agilent J&W) at 250 °C and then separated with helium as the mobile phase. The column was maintained at 50 °C for 1 minute after injection and then eluted by a temperature gradient to 300 °C at a rate of 10 °C min^{-1}.

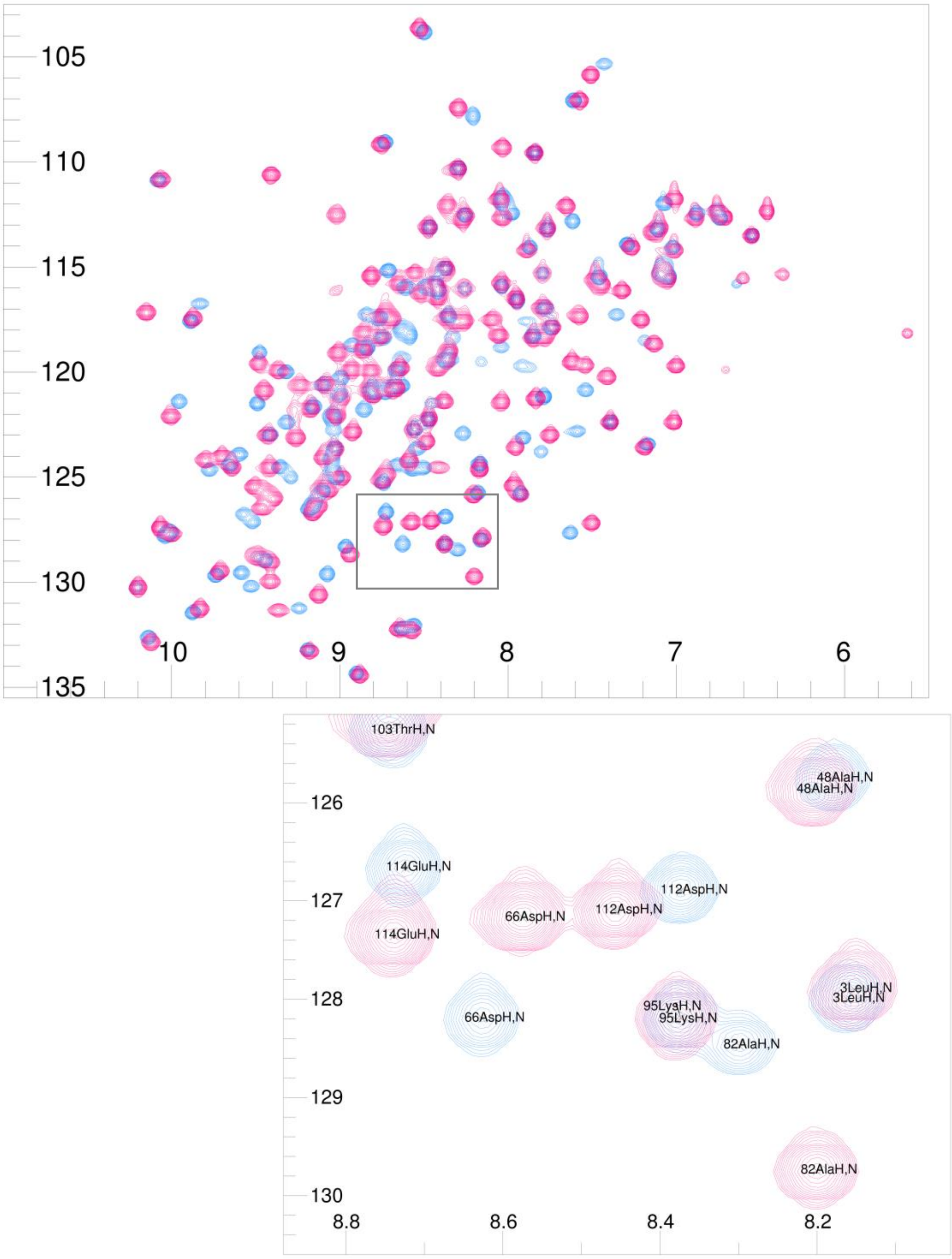

Figure S4. A. Overlaid ^{15}N HSQC NMR spectra of As-p18 protein with (blue) and without (pink) oleate. The with oleate sample was prepared with a protein:ligand ratio of 1:1.5. B. Inset showing chemical shift changes at selected amino acid positions affected by the presence of ligand.

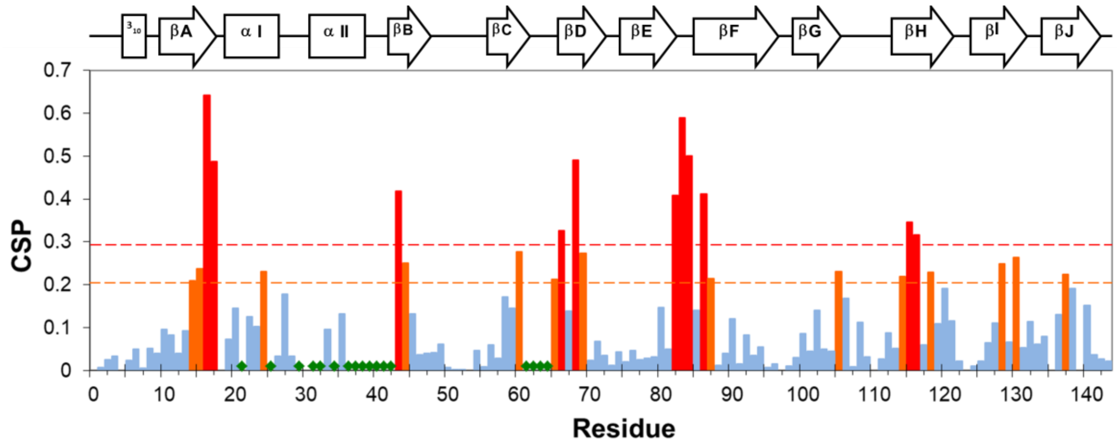

Figure S5. Histogram of the chemical shift perturbations (CSP = $[(\Delta\delta^1H)^2 + (0.2 \times \Delta\delta^{15}N)^2]^{1/2}$) induced in the backbone amides of As-p18 by oleate binding. Red and orange dotted lines indicate the two thresholds (average CSP plus two or one standard deviation respectively). Green diamonds are displayed for each of the residues whose peaks are not observed in the *apo* form. The locations of regular secondary structure elements are indicated by the cartoon above the graph.

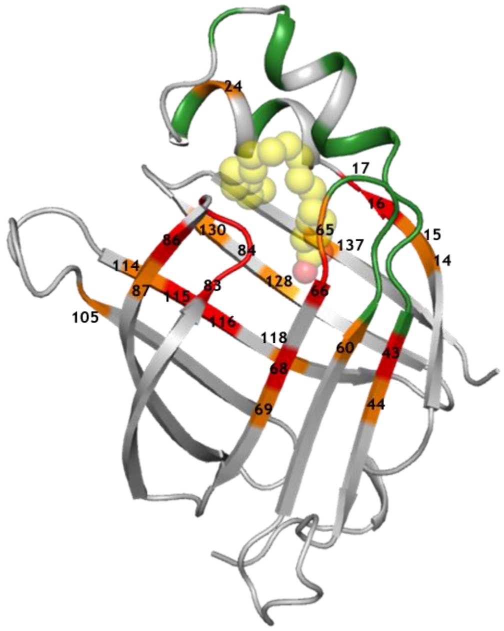

Figure S6. Positions of amino acids in As-p18 that undergo the greatest chemical shift perturbations (CSPs) between *apo* and *holo* forms. As-p18 is shown as a backbone cartoon with residues that experience CSPs greater than 1 or 2 σ coloured orange and red respectively. Residues 21, 25, 29, 31, 32, 34, 36-42 & 61-64, whose amides are only visible in the holo form are shown in green. Oleic acid is depicted as yellow (carbon) and red (oxygen) spheres.

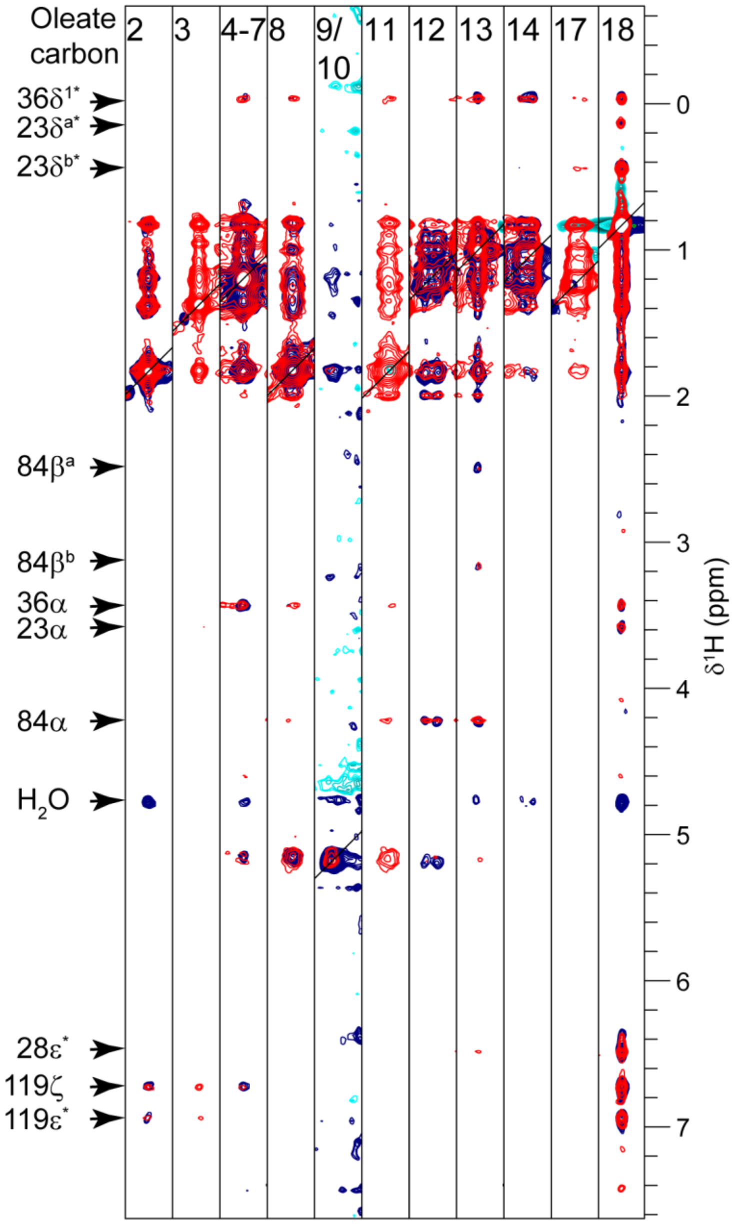

Figure S7. Intermolecular NOEs observed in samples of unlabelled As-p18 loaded with (U-^{13}C-)oleate (red) or (2,4,6,8,10,12,14,18-^{13}C-)oleate (dark blue/cyan). The strips shown are extracted from 3D, ^{13}C-edited NOESY spectra of each complex. Each strip is centred on the ^{13}C chemical shift of the indicated oleate carbon (or carbons) in the axis perpendicular to the page, and on the ^{1}H shift(s) of its (or their) attached hydrogen atoms in the horizontal axis. Correlated As-p18 resonances are indicated at the left hand side with the most likely assignment shown.

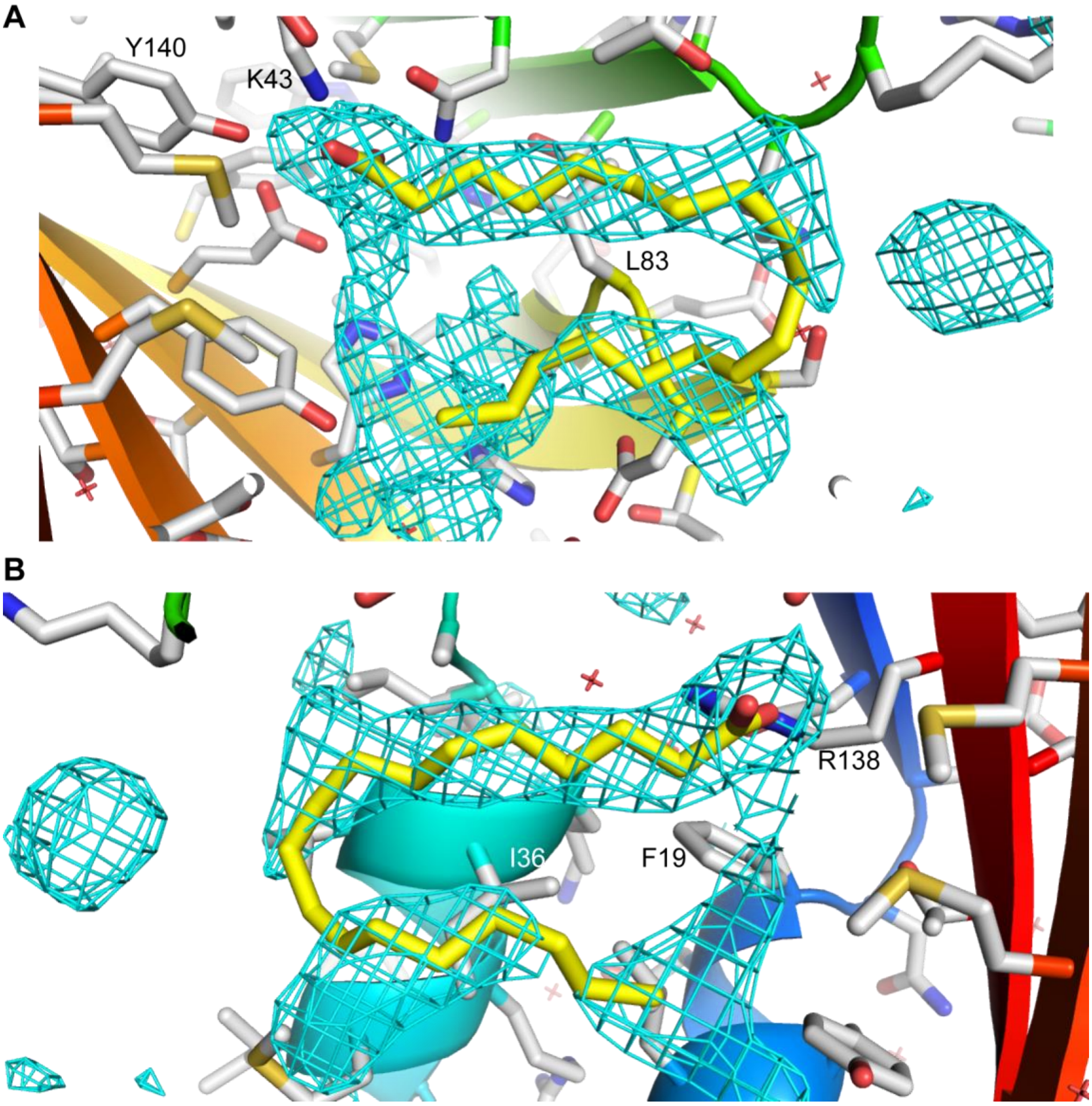

Figure S8. Electron density in the ligand binding site of unstripped As-p18. The polder OMIT map for one copy of As-p18 in the asymmetric unit is shown countoured at 3σ (blue mesh) with the protein shown as a backbone cartoon coloured from blue (N-terminal) to red (C-terminal) and with its sidechains shown as sticks with non-Cα atoms coloured by atom type (oxygen: red; nitrogen: blue; carbon: grey). Vaccenic acid is shown in yellow sticks with oxygen atoms coloured red. Selected residues that may close contact with the vaccenic acid are labelled. Views A and B are related by a 180° rotation about a vertical axis and the z-slice chosen so that protein residues do not obscure the view of the ligand.
